# Identification of *Mycobacterium avium* subspecies *paratuberculosis* in sheep farms in Bayannaoer, Inner Mongolia, China (short communication)

**DOI:** 10.1186/s12917-022-03293-6

**Published:** 2022-07-16

**Authors:** Yuandi Yu, Suhui Zhang, Guoyang Xu, Dengfeng Xu, Hua Zheng, Bo Li, Kefei Shen, Lizhi Fu

**Affiliations:** 1 Chongqing Academy of Animal Sciences, Chongqing, China; 2 Chongqing Research Center of Veterinary Biologicals Engineering and Technology, Chongqing Academy of Animal Sciences, 51 Changlong Avenue, Rongchang District, ChongQing, 402460 China

**Keywords:** Sheep, Paratuberculosis, ELISA, PCR, Histopathology

## Abstract

**Background:**

Paratuberculosis is a widespread chronic infection of *Mycobacterium avium* subspecies *paratuberculosis* (MAP) that causes significant economic losses to the sheep industry. The current study investigated this disease, which causes diarrhea in sheep, particularly, in Bayannaoer, Inner Mongolia, China. Diagnosis was based on clinical symptoms, pathological autopsy, histopathological inspection, and serological and molecular methods.

**Results:**

MAP was confirmed using polymerase chain reaction using DNA extracted from tissue and fecal samples. Serum samples from 472 individual sheep were obtained to detect antibodies against MAP using an enzyme-linked immunosorbent assay. MAP antibodies were separately detected in 17.86% (35/196) and 18.48% (51/276) of sheep herds at approximately 6 months and ≥ 1 year of age, respectively. The tissue lesion and pathological section results were consistent with *paratuberculosis* infection.

**Conclusions:**

To our knowledge, this is the first report of *Mycobacterium avium* subspecies *paratuberculosis* seroprevalence in Bayannaoer sheep in Inner Mongolia. Our findings show that MAP is not only prevalent, but also a potential threat to this region. Further investigations, including long-term epidemiological surveillance and isolation are needed for the awareness and effective treatment of paratuberculosis in sheep of Inner Mongolia.

## Background

Johne’s disease (JD) or paratuberculosis (PTB) is a chronic, progressive granulomatous enteritis disease that is caused by the zoonotic bacterium, *Mycobacterium avium* subspecies *paratuberculosis* (MAP) [1]. The disease primarily affects ruminants, including cattle, sheep, and goats. *Paratuberculosis* is a public health threat, and it reduces animal productivity and causes significant economic losses in ruminant industries worldwide [2, 3]. The common clinical manifestations are chronic diarrhea or scours, decreased productivity, weight loss and eventually, death of the animal [4]. Many countries have reported that infection with JD in sheep and goats occurs in a more discrete and insidious manner than in other animals. In Europe, the infection rate in sheep and goat herds is approximately 4% [5, 6]. Furthermore, in some Australian flocks, mortality rate reached 20% per annum [7]. An economic study showed that MAP infection reduced the profit efficiency from 84% to 64% in Italian dairy sheep and goat farms [8]. Other countries and regions have also shown varying degrees of economic losses as a result of PTB [9].

In recent years, MAP infection of ruminants in China has occurred rapidly. Some studies have indicated that MAP has become a common pathogen in dairy farms. Notably, the herd-level prevalence of 57.9% in 19 dairy herds in the Shandong province of China was observed [10]. MAP infection in dairy cattle differs with farming modes at the animal and herd level, and farming density could be an important risk factor associated with the presence of MAP-infected cattle [11]. One study showed that the overall MAP seroprevalence in the tested Tibetan sheep was 11.29% [12]. Additionally, recent studies have shown that PTB is widely prevalent in dairy farms in Tai'an, in the Shandong province of China [13]. Therefore, it is evident that research should focus on the prevalence, prevention, and control of PTB.

Moreover, the mutton sheep industry plays an important role in Bayannaoer, which is in western Inner Mongolia in China. Every year, approximately 10% of sheep in this area have intermittent diarrhea, against which pharmaceutical treatments are ineffective. This results in the eventual death of these sick sheep due to exhaustion, which emphasizes the significant negative effect that PTB has on economic development. As such, we diagnosed several sheep herds with PTB through clinical inspection, molecular biological methods, histopathological examination, and serological detection, to understand the epidemiology of MAP in Bayannaoer.

## Results

### Clinical symptoms and necropsy findings

**Fig. 1:**
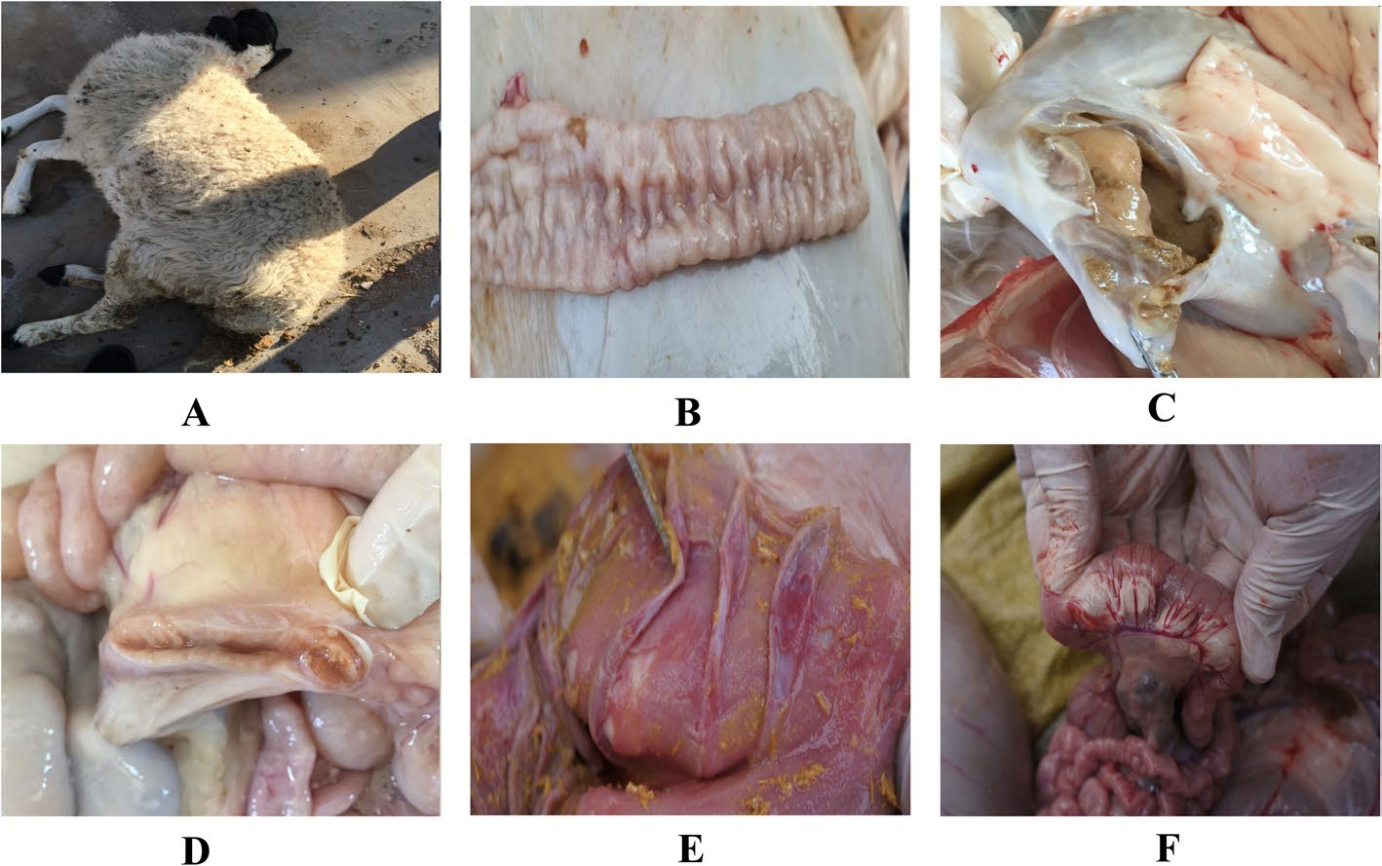
Symptoms and Necropsy of Mycobacterium avium subsp. paratuberculosis infected Sheep. **A** Sheep with signs of diarrhea and emaciation. **B** Thickening and corrugation of the intestinal mucosa. **C** Enlarged and edematous abomasum. **D** Mesenteric lymph node edema and liquefaction. **E** Intestinal mucosa exfoliation. **F** Mesenteric congestion

The sick sheep showed clinical signs of JD, including severe weight loss and chronic diarrhea (Fig. 1 A).

Necropsy findings in representative dead sheep showed ileal hyperplasia (Fig. 1 B), enlarged and edematous abomasum (Fig. 1C), in addition to mesenteric lymph node edema and liquefaction (Fig. 1D). Mesenteric congestion and intestinal mucosa exfoliation were also found in intestinal lesions (Fig. 1 E, and F).

### Polymerase chain reaction (PCR) MAP detection

**Fig. 2:**
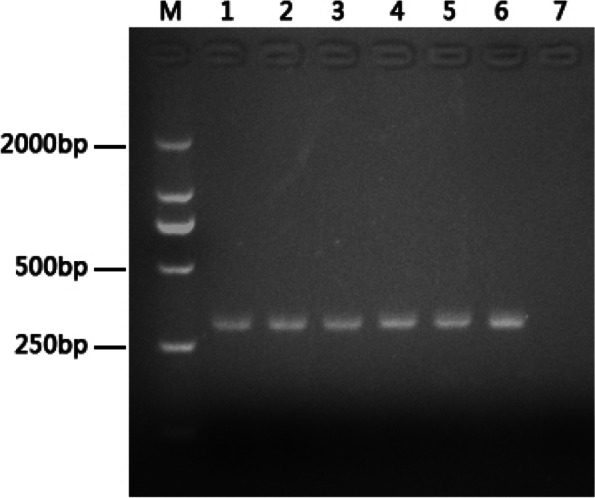
PCR products from the IS*900*[L/AV] nested PCR. M: DNA marker 2000 bp. Lane 1-Lane3: Positive sample of feces; Lane 4-Lane3: Positive sample of mesenteric lymph node samples; Lane 7: negative sample

The deoxyribonucleic acids (DNA) from fecal and ileum samples of sheep with diarrhea were used as templates. The results showed that the fragment size amplified by the nested PCR (L/AV) targeting IS900 was 298 bp, consistent with the expected size (Fig. 2).

### Incidence of MAP in sheep

A total of 472 serum samples collected from different sheep farms were tested using enzyme-linked immunosorbent assay (ELISA). The sheep were either approximately 6 months or ≥ 1 year old, and the detection rates of sheep in these two stages were similar. A total of 17.86% (35/196) and 18.48% (51/276) were separately tested positive for MAP in the two age groups (Table 1).

**Table 1:** The total number (n) of animals at two different age groups (6 months and ≥ 1 year old) with the number of positive MAP samples, and the positive MAP detection rate (%) sampled from each herd in Bayannaoer, Inner Mongolia, China

Herds	Sampling Number		Number of MAP-positive samples		Positive MAP detection rate (%)	
	Approx. 6 months old	≥ 1 year old	Approx. 6 months old	≥ 1 year old	About 6 months old	≥ 1 year old
A	11	16	3	4	27.27	25
B	18	25	4	5	22.22	20
C	20	27	5	6	25	22.22
D	12	17	3	4	25	23.53
E	16	22	1	2	6.25	9.09
F	19	24	4	6	21.05	25
G	14	20	2	3	14.29	15
H	13	19	4	5	30.77	26.32
I	9	14	2	3	22.22	21.43
J	15	23	0	2	0	8.70
K	21	29	3	5	14.29	17.24
L	10	15	0	1	0	6.66
M	18	25	4	5	22.22	20

### Histopathological assessment

**Fig. 3:**
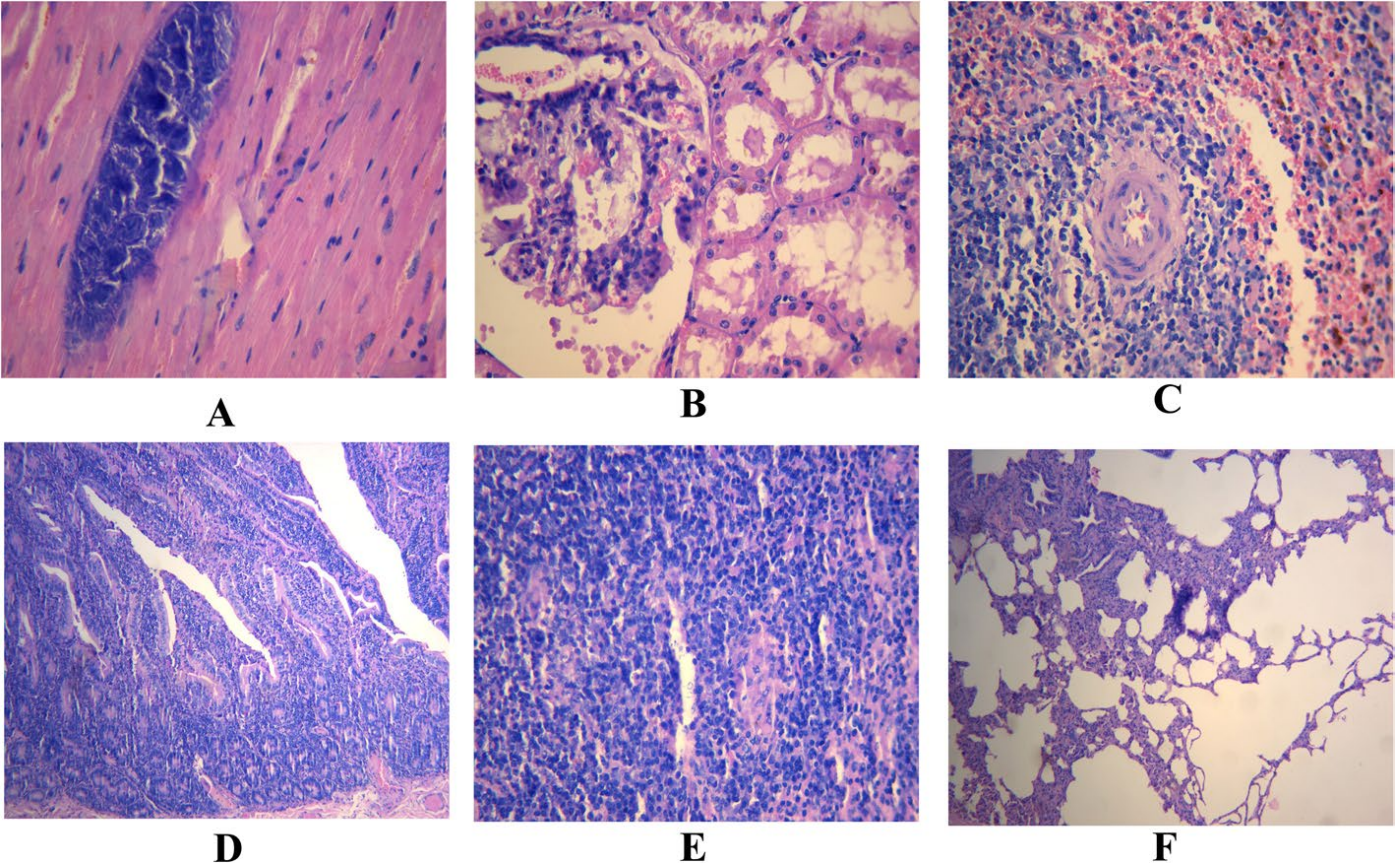
Pathological changes of the diseased sheep. **A** Myocardial fiber swelling, thickening and calcification. **B** Renal tubulointerstitial injuries . **C** Massive necrosis of spleen lymphocytes. **D** Thickening of the intestinal mucosa. **E** Necrosis of mesenteric lymph nodes. **F** Pathological changes of lung tissues

The heart, spleen, lung, kidney, intestinal, and mesenteric lymph nodes of the sheep were stained with hematoxylin and eosin (H & E) to detect MAP infection. The myocardial fibers were swollen, thickened, and partially broken, and the transverse striations were not obvious. Moreover, there were scattered calcifications of different sizes (Fig. 3A). The endothelial cells of glomerular capillaries were partially necrotic, with red-stained silk reticular fibrin and a small amount of red blood cells distributed between them; a small amount of red blood cells was also seen in the renal capsule. In addition, the epithelial cells of the renal tubules were swollen and partially necrotic, and protein tubules appeared in most of the lumens (Fig. 3B). Many spleen lymphocytes were necrotic and significantly bleeding, especially around the splenic nodules, forming a red halo (Fig. 3C). The intestinal mucosa was thickened; many inflammatory cells were distributed in the lamina propria (Fig. 3D), and some lymphocytes in the mesenteric lymph nodes were necrotic and scattered in granulomas of different sizes. Moreover, epithelioid cells and lymphocytes were observed in these granulomas (Fig. 3E). The pulmonary interstitium widened, and many inflammatory cells were distributed. Most of the alveoli were dilated, and the alveolar wall ruptured (Fig. 3F).

## Discussion

Johne’s disease is one of the most economically important diseases affecting ruminants worldwide, and it is a growing concern in the sheep and goat industries [14]. The disease is caused by the zoonotic pathogen MAP, that poses a challenge to public health [3]. The sheep farming and industrial wool spinning industries are particularly developed in Bayannaoer. Therefore, MAP could act as a threat to workers in these industries and to public health security. In 2011, the flocks in this region suffered from intermittent diarrhea and eventually died of exhaustion. Most of them were adult sheep, and the main symptoms were similar to those reported in previous studies—elevated body temperature, dark green and thick feces [15, 16]. The course of the disease was 10–30 days, over which the sick sheep gradually lost weight and their eyelids became white. This brought huge economic losses to local herdsmen; therefore, it is essential to control the spread of this disease. However, clinical signs in sheep or goats are not a reliable indicator of the presence or absence of MAP infection [17]. Weight loss is the predominant clinical sign in infected sheep and goats. In sheep, the period of weight loss differs from one animal to another. Softening of the faeces or diarrhoea occurs only in 20% of the cases at the end stages of the disease [18]. Detailed epidemiological investigations are important to understand the regional prevalence and potential threats of MAP. MAP can also induce bacteremia, which was unevenly distributed in the filtering organs of the sheep [19]. Attention should be paid to MAP infection in young sheep. Two clinical syndromes have been described in red deer: sporadic disease with low morbidity and high mortality in adult populations, and severe outbreaks in young deer (8–15 months old) resulting in both high morbidity and high mortality [20]. Young animals, especially those under 6 months, have a higher risk of infecting MAP, but the risk decreases thereafter [21]. Research also shows that calves inoculated with MAP at an earlier age had more severe tissue lesions [22].

In this study, we provided a detailed description of the clinical inspection and pathological changes, as well as molecular biology techniques and ELISA to diagnose naturally occurring PTB cases. We found that approximately 10% of sheep were infected in February and March; notably, most infections occurred in August and fewer infections were observed in winter. This cycle is almost the same every year. The main symptoms were emaciation and general weakness with intermittent diarrhea in infected sheep, which is consistent with the infection of paratuberculosis [18].

Paratuberculosis was confirmed by PCR using DNA extracted from mesenteric lymph node tissues and feces. At the histopathological level, the tissues and organs of the sheep, in this study, showed different degrees of lesions at the late stage of the disease. Serum samples of different sheep flocks were subjected to a MAP ELISA, and the seroprevalence was found to be 17.86% (35/196) and 18.48% (51/276), respectively, for the two different age groups. This prevalence was lower than the 19.5% seroprevalence in sheep in the Eastern Province of Saudi Arabia [23]; however, it was higher than the 11.9% seroprevalence in the Tibetan sheep in China [12], and 3.25% in female sheep in Tunisia [24]. These data show that PTB may be a risk factor in Inner Mongolia; however, detailed statistical analysis is needed.

MAP causes chronic diarrheic intestinal infections, which are difficult to treat, in domestic animals, including ruminants [25]. Several aminoglycosides are active against several species of mycobacteria, including MAP [26]. In this study, gentamicin was used in combination with lincomycin to treat diseases associated with MAP. We found that the symptoms in sheep were relieved after treatment for the first time; however, diarrhea occurred approximately 30 days after the first treatment. At this time, the use of therapeutic drugs was ineffective, and the sick sheep continued to suffer from diarrhea and subsequently died of exhaustion. The results demonstrated that some products effectively inhibited bacterial growth, and since these findings are applicable to the veterinary field, these products may become available for veterinary use [27, 28]. Further research may substantiate the efficacy of pharmaceuticals for the treatment and control of PTB.

## Conclusion

In conclusion, this study is the first to estimate the epidemiology of PTB in sheep in Bayannaoer, Inner Mongolia, China. Our results showed that MAP was prevalent in this region. This information will provide a comprehensive view for the prevention and control of MAP infection in Inner Mongolia sheep.

## Methods

### Sampled animals and Sample collection

This study was carried out during October 2020, a total number of 472 sheep from 13 small to middle-sized sheep farms were sampled. The sampling proportion of the sheep was not less than 50% for the 6-month old sheep, and the proportion of sheep over 1 year old was not more than 5%. In addition, 196 sheep at approximately 6 months old and 276 sheep at ≥ 1 year old were selected. Sheep owners were interviewed about all treatments, therapeutic agents, and incidences of MAP in their sheep flocks. In this study, aminoglycosides were used to treat the disease, such as intramuscular injection of gentamicin (4 mg/kg) combined with oral electrolytic multidimensional preparation and lincomycin (500 mg oral), twice a day for 3 consecutive days. Three sheep that were extremely weak and were suspected of having JD were euthanized. The study was in compliance with ARRIVE guidelines. Serum and tissues samples, including ileum and mesenteric lymph node samples, were collected. Three milliliters of blood were collected from the jugular vein of each animal using a vacutainer. Sera were collected in Eppendorf tubes and stored at –20 °C until use [24]. Necropsy findings of the carcasses showed emaciated intestinal mucosa, enlarged lymph nodes, and occasional serous fat atrophy. The samples were separated into two parts, immediately placed in sterile plastic bags, placed in cooling boxes, and transported to the microbiology laboratory. Samples of the carcasses were subjected to tissue staining and DNA extraction.

### PCR detection of MAP

DNA was extracted from the feces and mesenteric lymph node samples of sick sheep and subjected to PCR for MAP detection. DNA was extracted from fecal samples using the QIAamp Fast DNA Stool Mini Kit (Qiagen, Hilden, Germany) and from tissue using the TIANamp Genomic DNA Kit (Tiangen, Beijing, China). The primers targeting IS900 were designed as follows: L1 (5’-CTTTCTTGAAGGGTGTTCGG-3’) and L2 (5’ -ACGTGACCTCGCCTCCAT-3’), AV1(5’-ATGTGGTTGCTGTGTTGGATGG-3’) and AV2 (5’-CCGCCGCAATCAACTCCAG-3’) and were used for the first round of PCR and nested PCR, respectively, as previously described [29]. For the first round of PCR, conditions were as follows: 94°C for 2 min, followed by 30 cycles at 94°C for 30 s, 58°C for 30 s, and 72°C for 30 s, with a final elongation step at 72°C for 3 min. The second PCR round used a 10× dilution of the products of the first round as templates, and the reaction parameters were carried out according to the primary reaction.

### ELISA assays

Serum samples were collected from sheep farms in Bayannaoer, with herd sizes ranging from 300 to 500. The samples were analyzed using a commercial indirect ELISA kit. The tests were performed according to the manufacturer’s instructions (ID-VET, Montpellier, France). Sera with sample to positive (S/P) ratios ≤ 75% were scored as MAP-negative, while those with ratios ≥ 85% were considered MAP-positive. Moreover, 75%< S/P% <85% were scored as “suspect” and treated as negative for data analysis.

### Histopathological examination

Tissue samples from the heart, kidney, spleen, lungs, ileum and mesenteric lymph nodes were fixed in 10% neutral buffered formalin, embedded in paraffin, sectioned at 5-μm thickness and stained with H & E.

## Data Availability

All data generated or analyzed during this study are included in this published article.

## Abbreviations

DNA: Deoxyribonucleic acid
ELISA: Enzyme linked immunosorbent assay
JD: Johne’s disease
MAP: *Mycobacterium avium* subspecies paratuberculosis
PCR: Polymerase Chain Reaction
PTB: Paratuberculosis
